# Supplementary material for: Tead1a Initiates Transcriptional Priming Through the TEAD1a/YAP‐Notch1‐Spi1/Cebpα Axis to Promote Neutrophil Fate
Source: Adv Sci (Weinh). 2025 Aug 28;12(41):e05441. doi: 10.1002/advs.202505441 (PMC12591127; doi:10.1002/advs.202505441)
Supplement: Supplementary file 4 — Supplementary Dataset [file ADVS-12-e05441-s004.zip › 3-1.zip!/3-1/02-1.cluster/cluster1/Enrich/ego_CC.pdf]

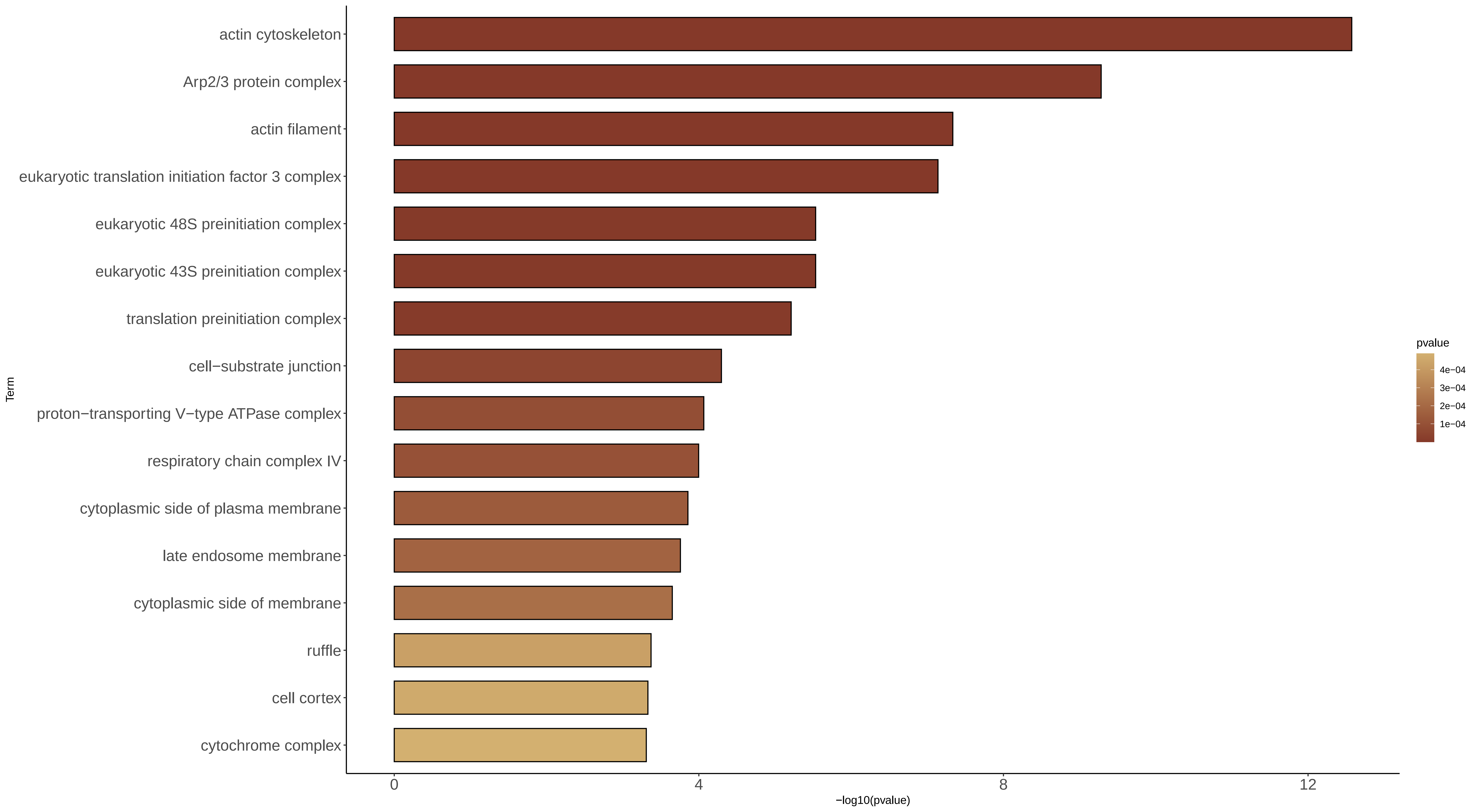
actin cytoskeleton
Arp2/3 protein complex
actin filament
eukaryotic translation initiation factor 3 complex
eukaryotic 48S preinitiation complex
eukaryotic 43S preinitiation complex
translation preinitiation complex
cell−substrate junction
proton−transporting V−type ATPase complex
respiratory chain complex IV
cytoplasmic side of plasma membrane
late endosome membrane
cytoplasmic side of membrane
ruffle
cell cortex
cytochrome complex
Term
0
4
8
12
−log10(pvalue)
pvalue
4e−04
3e−04
2e−04
1e−04
